# The Landscape of Virus-Host Protein–Protein Interaction Databases

**DOI:** 10.3389/fmicb.2022.827742

**Published:** 2022-07-15

**Authors:** Gabriel Valiente

**Affiliations:** Algorithms, Bioinformatics, Complexity and Formal Methods Research Group, Department of Computer Science, Technical University of Catalonia, Barcelona, Spain

**Keywords:** protein–protein interaction, virus-host protein–protein interaction, protein–protein interaction database, virus-host protein–protein interaction database, overlap

## Abstract

Knowledge of virus-host interactomes has advanced exponentially in the last decade by the use of high-throughput screening technologies to obtain a more comprehensive landscape of virus-host protein–protein interactions. In this article, we present a systematic review of the available virus-host protein–protein interaction database resources. The resources covered in this review are both generic virus-host protein–protein interaction databases and databases of protein–protein interactions for a specific virus or for those viruses that infect a particular host. The databases are reviewed on the basis of the specificity for a particular virus or host, the number of virus-host protein–protein interactions included, and the functionality in terms of browse, search, visualization, and download. Further, we also analyze the overlap of the databases, that is, the number of virus-host protein–protein interactions shared by the various databases, as well as the structure of the virus-host protein–protein interaction network, across viruses and hosts.

## 1. Introduction

Knowledge of virus-host interactomes has advanced exponentially in the last decade by the use of high-throughput screening technologies to obtain a more comprehensive landscape of virus-host protein–protein interactions (de Chassey et al., 2014; Sharma et al., 2015). Beyond physical methods such as affinity chromatography and coimmunoprecipitation (Phizicky and Fields, 1995), the development of mass spectrometric methods such as the yeast two-hybrid system (Fields and Sternglanz, 1994) and affinity purification combined with mass spectrometry (Kim et al., 2010) has fostered the high-throughput identification and characterization of protein–protein interactions (Börnke, 2008), computationally predicted and experimentally validated using these techniques, for protein–protein interactions within single bacteria, viruses, and small and large eukaryotes (Zhang, 2009) and also for interactions between viral proteins and proteins of the host they infect (Brito and Pinney, 2017).

In this article, we present a systematic review of the available virus-host protein–protein interaction database resources. The resources covered in this review are seven generic virus-host protein–protein interaction databases: EBI-GOA-nonIntAct (Huntley et al., 2015), BioGRID (Oughtred et al., 2021), VirusMentha (Calderone et al., 2015), IntAct (Orchard et al., 2014), VirHostNet (Navratil et al., 2009; Guirimand et al., 2015), HPIDB (Kumar and Nanduri, 2010), and Viruses.STRING (Cook et al., 2018), as well as one database of protein–protein interactions for a specific virus, HCVpro (Kwofie et al., 2011), and three databases of protein–protein interactions for those viruses that infect a particular host, VirusMINT (Chatr-aryamontri et al., 2009), PHISTO (Tekir et al., 2013), and HVIDB (Yang et al., 2021).

The databases are reviewed on the basis of the specificity for a particular virus or host, the number of virus-host protein–protein interactions included, and the functionality in terms of browse, search, visualization, and download. Further, we also analyze the overlap of the databases, that is, the number of virus-host protein–protein interactions shared by the various databases, as well as the structure of the virus-host protein–protein interaction network, across viruses and hosts.

## 2. Methods and Results

### 2.1. Databases

For all the generic databases, we downloaded the virus-host protein–protein interaction data. The current (October 2021) release of EBI-GOA-nonIntAct, downloaded from http://www.ebi.ac.uk/Tools/webservices/psicquic/view/, contained 18,468 unknown, 105 virus-virus, 1,009 virus-host, and 77,852 host-host protein–protein interactions. Release 4.4.202 of BioGRID, downloaded from https://downloads.thebiogrid.org/BioGRID/Release-Archive/BIOGRID-4.4.202/, contained 702 virus-virus, 28,473 virus-host, and 2,256,186 host-host protein–protein interactions. The August 2021 update of VirusMentha, downloaded from https://virusmentha.uniroma2.it/, contained 10,907 virus-host protein–protein interactions. The current (October 2021) release of IntAct, downloaded from http://ftp.ebi.ac.uk/pub/databases/intact/current/psimitab/intact-micluster.zip, contained 18,468 unknown, 2,680 virus-virus, 26,443 virus-host, and 621,788 host-host protein–protein interactions. The March 2021 release of VirHostNet, downloaded from https://virhostnet.prabi.fr/, contained 4,442 virus-virus, 35,405 virus-host, and 158 host-host protein–protein interactions. The current (August 2021) release of HPIDB, downloaded from https://hpidb.igbb.msstate.edu/, contained 51,216 virus-host and 18,571 host-host protein–protein interactions. Last, release 10.5 of Viruses.STRING, downloaded from http://viruses.string-db.org/, contained 12,420 virus-virus, 330,136 virus-host, and 650,750,772 host-host protein–protein interactions. The ETE3 toolkit (Huerta-Cepas et al., 2016) version 3.1.2 was used to map the taxonomic identifiers for the proteins to the NCBI Taxonomy (Schoch et al., 2020) in order to determine their classification as virus or host proteins.

We also downloaded the virus-host protein–protein interaction data for all the virus-specific and host-specific databases. The current (October 2021) release of HCVpro, downloaded from https://www.cbrc.kaust.edu.sa/hcvpro/, contained 621 virus-host protein–protein interactions. The current (October 2021) release of VirusMINT, from https://maayanlab.cloud/Harmonizome/dataset/Virus+MINT+Protein-Viral+Protein+Interactions, contained 1,036 virus-host protein–protein interactions. The current (October 2021) release of PHISTO, downloaded from https://phisto.org/, contained one unknown and 52,976 virus-host protein–protein interactions. The current (October 2021) release of HVIDB, downloaded from http://zzdlab.com/hvidb/, contained 48,643 virus-host protein–protein interactions.

**Figure 1:**
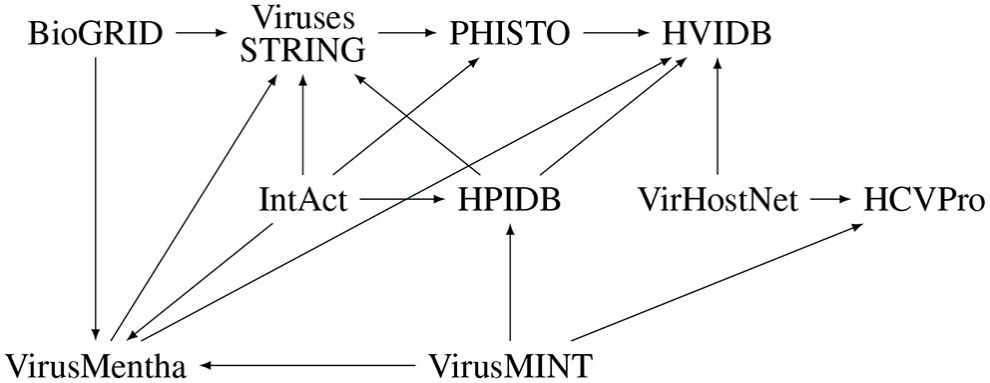
Dependencies among virus-host protein–protein interaction databases.

EBI-GOA-nonIntAct, BioGRID, IntAct, VirHostNet, HPIDB, and VirusMINT contain interactions derived from literature curation which are, in most cases, experimentally validated virus-host protein–protein interactions, while VirusMentha, HPIDB, Viruses.STRING, HCVpro, PHISTO, and HVIDB essentially integrate virus-host protein–protein interactions from other databases. In fact, VirusMentha takes virus-host protein–protein interactions from VirusMINT, IntAct, DIP (Salwinski et al., 2004), MatrixDB (Chautard et al., 2011), and BioGRID; HPIDB takes interactions from BIND (Alfarano et al., 2005), VirusMINT, PIG (Driscoll et al., 2009), GeneRIF (Jimeno-Yepes et al., 2013), Reactome (Croft et al., 2011), and IntAct; Viruses.STRING takes interactions from BioGRID, IntAct, DIP, HPIDB, and VirusMentha; HCVpro takes interactions from BIND, VirusMint, and VirHostNet; PHISTO takes interactions from APID (Prieto and De Las Rivas, 2006), IntAct, DIP, VirusMINT, iRefIndex (Razick et al., 2008), Viruses.STRING, MPIDB (Goll et al., 2008), BIND, and Reactome; and HVIDB takes virus-host protein–protein interactions from VirusMentha, VirHostNet, HPIDB, PHISTO, and PDB (Rose et al., 2017). Despite these dependencies among the databases, further illustrated in Figure 1, there is not much overlap among them, as discussed in Section 2.5.

These databases were chosen by means of a comprehensive literature search, and complemented with suggestions by the reviewers. P-HIPSTer (Lasso et al., 2019) was discarded because, unfortunately, the 282,528 computationally predicted viral-human protein–protein interactions therein are not available for download. ViRBase (Li et al., 2015) was discarded because the virus-host interactions therein are ncRNA-associated interactions, not protein–protein interactions and, in fact, none of the 44,276 gene symbols or 56,678 miRBase identifiers in ViRBase version 3.0 could be mapped to UniProtKB-AC unique identifiers.

### 2.2. Datasets

In order to be able to analyze the overlap of the databases, we mapped all virus and host protein identifiers to UniProtKB-AC unique identifiers, using the programmatic access to the database identifier mapping service at https://www.uniprot.org/mapping/. Host protein identifiers in the Viruses.STRING database were also mapped to UniProtKB-AC unique identifiers using the mapping files available at https://version-10-5.string-db.org/mapping_files/uniprot_mappings/. Apart from discarding any virus-virus and host-host protein–protein interactions, some of the virus-host protein–protein interactions had to be discarded as well, because the corresponding virus or host protein identifiers could not be mapped to UniProtKB-AC in a unique way. We have also raised viral strains to the species (virus) level, in order to facilitate comparison of virus-host protein–protein interactions across the databases. The resulting virus-host protein–protein interaction datasets are summarized in Table 1 and further detailed below.

**Table 1:** Virus-host protein–protein interaction datasets with UniProtKB-AC unique identifiers.

**Database**	**Viruses**	**Hosts**	**Viral proteins**	**Host proteins**	**Interactions**
EBI-GOA-nonIntAct	77	26	173	455	534
BioGRID	13	6	50	2,101	5,157
VirusMentha	114	8	627	3,624	10,626
IntAct	197	68	1,062	8,102	22,727
VirHostNet	128	6	984	7,361	28,132
HPIDB	205	36	1,387	7,570	33,906
Viruses.STRING	186	61	1,703	52,440	242,784
HCVpro	1	1	7	138	140
VirusMINT	28	1	287	287	372
PHISTO	182	1	1,700	6,520	39,010
HVIDB	146	1	1,313	7,060	40,132

The 1,009 virus-host protein–protein interactions in the EBI-GOA-nonIntAct database contained 628 UniProtKB AC/ID identifiers, all of which were mapped to UniProtKB-AC in a unique way. This resulted in 534 unique virus-host protein–protein interactions among 173 unique proteins from 77 viruses and 455 unique proteins from 26 hosts.

The 28,473 virus-host protein–protein interactions in the BioGRID database contained 4 UniProtKB AC/ID identifiers, all of which were mapped to UniProtKB-AC in a unique way; 2 BioGRID identifiers, which could not be mapped to UniProtKB-AC; and 6,589 Entrez Gene (GeneID) identifiers, 3,007 of which were mapped to UniProtKB-AC in a unique way. This resulted in 5,157 unique virus-host protein–protein interactions among 50 unique proteins from 13 viruses and 2,101 unique proteins from 6 hosts.

The 10,907 virus-host protein–protein interactions in the VirusMentha database contained 4,347 UniProtKB AC/ID identifiers, 4,332 of which were mapped to 4,313 UniProtKB-AC in a unique way. This resulted in 10,626 unique virus-host protein–protein interactions among 627 unique proteins from 114 viruses and 3,624 unique proteins from 8 hosts.

The 26,443 virus-host protein–protein interactions in the IntAct database contained 10,282 UniProtKB AC/ID identifiers, 10,047 of which were mapped to UniProtKB-AC in a unique way. This resulted in 22,727 unique virus-host protein–protein interactions among 1,062 unique proteins from 197 viruses and 8,102 unique proteins from 68 hosts.

The 35,405 virus-host protein–protein interactions in the VirHostNet database contained 10,049 protein identifiers: 9,868 UniProtKB AC/ID identifiers, 9,717 of which were mapped to UniProtKB-AC in a unique way; 180 RefSeq Protein identifiers, 169 of which were mapped to UniProtKB-AC in a unique way; and one EMBL/GenBank/DDBJ identifier, which could not be mapped to UniProtKB-AC. This resulted in 28,132 unique virus-host protein–protein interactions among 984 unique proteins from 128 viruses and 7,361 unique proteins from 6 hosts.

The 51,216 virus-host protein–protein interactions in the HPIDB database contained 19,784 protein identifiers: 16,465 UniProtKB AC/ID identifiers, 16,295 of which were mapped to UniProtKB-AC in a unique way; 3,106 Entrez Gene (GeneID) identifiers, 1,928 of which were mapped to UniProtKB-AC in a unique way; 110 RefSeq Protein identifiers, 86 of which were mapped to UniProtKB-AC in a unique way; four EMBL/GenBank/DDBJ identifiers, one of which was mapped to UniProtKB-AC in a unique way; two Ensembl Protein identifiers, one of which was mapped to UniProtKB-AC in a unique way; one Ensembl Genomes Protein identifier, which was mapped to UniProtKB-AC in a unique way; and 96 IntAct identifiers, none of which could be mapped to UniProtKB-AC in a unique way. This resulted in 33,906 unique virus-host protein–protein interactions among 1,387 unique proteins from 205 viruses and 7,570 unique proteins from 36 hosts.

The 330,136 virus-host protein–protein interactions in the Viruses.STRING database contained 41,490 protein identifiers: 29,236 Ensembl Protein identifiers, 29,093 of which were mapped to UniProtKB-AC in a unique way; 1,371 Ensembl Genomes Protein identifiers, 1,212 of which were mapped to UniProtKB-AC in a unique way; and 131 UniProtKB AC/ID identifiers, all of which were mapped to UniProtKB-AC in a unique way. None of the remaining 10,752 identifiers could be mapped to UniProtKB-AC in a unique way. However, using the aforementioned mapping files, 37,395 host protein identifiers were mapped to UniProtKB-AC in a unique way. Combining the two approaches, this resulted in 242,784 unique virus-host protein–protein interactions among 1,703 unique proteins from 186 viruses and 52,440 unique proteins from 61 hosts.

The 621 virus-host protein–protein interactions in the virus-specific HCVpro database contained 487 protein identifiers, 145 of which were mapped to UniProtKB-AC in a unique way. This resulted in 140 unique virus-host protein–protein interactions among 7 unique Hepatitis C virus proteins and 138 unique human proteins.

The 1,036 virus-host protein–protein interactions in the host-specific VirusMINT database contained 706 gene identifiers and 706 protein identifiers. Only 993 of the 1,412 gene and protein identifiers were mapped to UniProtKB-AC in a unique way. This resulted in 391 unique virus-host protein–protein interactions among 287 unique proteins from 43 viruses and 287 unique human proteins.

The 52,976 virus-host protein–protein interactions in the host-specific PHISTO database contained 8,212 UniProtKB AC/ID identifiers, 8,167 of which were mapped to UniProtKB-AC in a unique way. This resulted in 39,010 unique virus-host protein–protein interactions among 1,700 unique proteins from 182 viruses and 6,520 unique proteins from one host.

Finally, the 48,643 virus-host protein–protein interactions in the host-specific HVIDB database contained 9,900 protein identifiers, 9,699 of which were mapped to UniProtKB-AC in a unique way. This resulted in 44,590 unique virus-host protein–protein interactions among 1,939 unique proteins from 737 viruses and 7,437 unique human proteins.

### 2.3. Functionality of the Databases

All the databases support, to some extent, browsing, searching, visualization, and download. While EBI-GOA-nonIntAct, BioGRID, VirusMentha, IntAct, and HPIDB only allow for browsing search results, VirHostNet allows for browsing the database by virus lineage (Baltimore class, family, species, and taxon) and by UniProtKB keyword annotation, and Viruses.STRING has no browsing facilities, although it allows for searching by virus or host name.

EBI-GOA-nonIntAct allows for searching over the entire database using a query language based on the PSI-MITAB format (Kerrien et al., 2007), using the PSICQUIC web service (del Toro et al., 2013). BioGRID allows for searching by gene name, publication identifier, and full text search using a simple query language. IntAct allows for searching by gene name, UniProtKB identifier, taxon identifier, publication identifier, and Gene Ontology terms. VirusMentha allows for searching by gene name, UniProtKB identifier, and keyword annotation, over the entire database or for a specific virus family or host. VirHostNet allows for searching by UniProtKB identifier, name, keyword annotation, virus lineage (species or taxon), and PubMed identifier (PMID), and also allows for BLASTP (Altschul et al., 1990) searches in a database of interacting protein sequences. HPIDB allows for regular expression searching by protein accession number or name, species or taxon identifier or name, PubMed identifier (PMID) or author name, and interaction type. Viruses.STRING allows for searching by protein, virus, and host name.

For the virus-specific and the host-specific databases, HCVpro allows for browsing by virus (Hepatitis C) protein name or host (human) protein name or chromosome, virus protein identifier, interaction type, and PMID, as well as for searching by host protein name or gene identifier. VirusMINT has no browse, search, or visualization facilities, as the resource at http://mint.bio.uniroma2.it/virusmint/ is no longer available. PHISTO allows for browsing by virus family and species, and searching by taxon identifier, virus name, virus or host protein name or UniProtKB identifier, experimental method, and PMID. HVIDB allows for browsing by viral family, and searching by UniProtKB identifier, UniProtKB entry name, gene identifier, gene name, protein name, and keyword annotation.

EBI-GOA-nonIntAct, BioGRID, VirusMentha, IntAct, VirHostNet, HPIDB, Viruses.STRING, PHISTO, and HVIDB all allow for visualization of search results using a graphics applet, Cytoscape.js (Franz et al., 2016) in the case of EBI-GOA-nonIntAct and VirHostNet. HCVpro has no such visualization facilities.

Download facilities differ among the various databases. For the generic databases, EBI-GOA-nonIntAct allows for downloading a single tab-separated (TSV) text file with all the interactions stored in the database, as the result of a query to the PSICQUIC web service. BioGRID allows for downloading a single text file, in PSI-MITAB format, with all the interactions stored in the database. VirusMentha allows for downloading a zip file containing a single semicolon-separated text file for each of the 8 hosts and for each of the 25 families of viruses covered in the database, and these zip files are updated every week. IntAct also allows for downloading a single text file in PSI-MITAB format with all the interactions stored in the database. VirHostNet also allows for downloading a single tab-separated text file with all the interactions stored in the database. HPIDB also allows for downloading a single text file in PSI-MITAB format with all the interactions stored in the database. Viruses.STRING allows for downloading a tar-gzip-compressed folder containing a single space-separated text file with either all the interactions stored in the database, or only those for a particular virus or host. On the other hand, for the virus-specific and the host-specific databases, all of them allow for downloading a single comma-separated (CSV) (for PHISTO) or tab-separated (for HCVpro, VirusMINT, and HVIDB) text file with all the virus-host interactions stored in the corresponding database. The main features of the various databases are summarized in Table 2.

**Table 2:** Main features of the virus-host protein–protein interaction databases.

**Database**	**Browse**	**Search**	**Visualization**	**Download**	**Update frequency**
EBI-GOA-nonIntAct	No	Yes	Cytoscape	TSV	Monthly
BioGRID	No	Yes	Yes	PSI-MITAB	Monthly
VirusMentha	No	Yes	Yes	CSV (semicolon)	Weekly
IntAct	No	Yes	Yes	PSI-MITAB	Every 8 weeks
VirHostNet	Yes	Yes	Cytoscape	TSV	Every 8 weeks
HPIDB	No	Yes	Yes	PSI-MITAB	Every 3 months
Viruses.STRING	No	Yes	Yes	CSV (space)	12 Aug 2021
HCVpro	Yes	Yes	No	TSV	Every 6 months
VirusMINT	No	No	No	TSV	26 Oct 2012
PHISTO	Yes	Yes	Yes	CSV	Monthly
HVIDB	Yes	Yes	Yes	TSV	25 Jun 2020

*Date of the last update is shown when the update frequency is unknown*.

### 2.4. Structure of the Virus-Host Protein–Protein Interaction Networks

The structure of biological networks in general, and protein–protein interaction networks in particular, can be analyzed by means of topological measures (Börnke, 2008; Steuer and López, 2008; Zhang and Hwang, 2009; Gaudelet and Pržulj, 2019; Hauschild et al., 2019). We show next that, under several of these topological measures, virus-host protein–protein interaction networks do not differ much from other protein–protein interaction networks.

Protein–protein interaction networks usually consist of a large component that fills most of the network, with the rest of the network divided into a large number of small components disconnected from the rest. Within each component, the average path length is the average length of the shortest paths for all pairs of nodes in the component. The average path length of a network is the average over all components of the average path length of each component, and average path lengths are usually small in biological networks (Newman, 2018).

Table 3 shows the size (number of nodes and edges), the number of connected components, the distribution of component sizes, and the average path length for the generic, virus-specific, and host-specific virus-host protein–protein interaction networks. These data show that virus-host protein–protein interaction networks also consist of a large component and a large number of small components, all of small average path length.

**Table 3:** Structure of the virus-host protein–protein interaction networks.

**Network**	**Nodes**	**Edges**	**Components**			**Average path length**
			**Number**	**Size**	**Count**	
EBI-GOA-nonIntAct	628	534	116	2–13	115	1.260108
BioGRID	2,151	5,157	5	2–3	4	1.636054
VirusMentha	4,252	10,625	69	2–45	68	1.273371
IntAct	9,164	22,677	145	2–55	144	1.306846
VirHostNet	8,345	28,132	35	2–8	33	1.208920
				8,147	1	
HPIDB	8,958	33,752	92	2–56	90	1.234933
				8,496	1	
Viruses.STRING	54,146	242,784	104	2–80	100	1.437420
				250	1	
				868	1	
				52,248	1	
HCVpro	145	140	5	2–4	4	1.366622
VirusMINT	659	372	287	2–8	287	1.073096
PHISTO	8,220	39,010	52	2–8	51	1.157549
HVIDB	8,373	40,132	26	2–7	25	1.293151

The degree of a node in a network is the number of edges attached to it, and the degree distribution of a network is the fraction *p*_*k*_ of the nodes that have degree *k*, for every *k*. Thus, *p*_*k*_ is the probability that a randomly chosen node in the network has degree *k*, and the degree distribution measures the frequency with which nodes of different degrees appear in the network (Newman, 2018).

**Figure 2:**
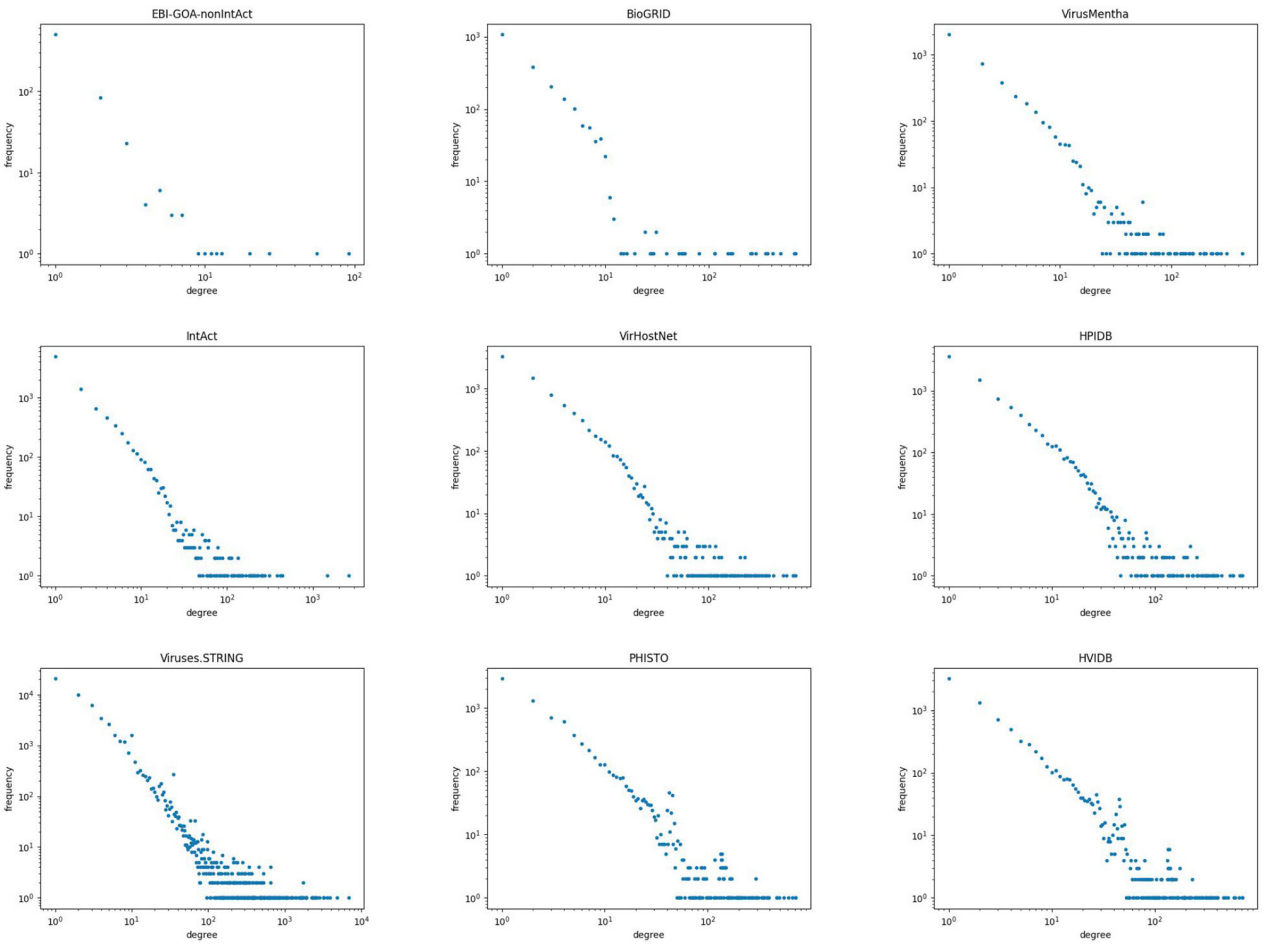
Degree distribution of the virus-host protein–protein interaction networks.

Biological networks tend to have degree distributions that follow a power law of the form $p_{k}\sim k^{-\gamma }$ for some positive constant γ, that is, a straight line with a negative slope. Figure 2 shows a scatter plot of the degree distribution, in logarithmic scale, for all but the two smallest virus-host protein–protein interaction networks. As can be seen therein, the degree distribution of virus-host protein–protein interaction networks follows a power law, that is, they are scale-free networks. The same behavior has been observed in other protein–protein interaction networks (Jeong et al., 2001; Barabási and Oltvai, 2004).

These structural properties of virus-host protein–protein interaction networks also characterize the networks for a specific virus or for the viruses that infect a specific host. Table 4 shows the size (number of nodes and edges), the number of connected components, the distribution of component sizes, and the average path length of the virus-host protein–protein interaction network for the *Influenza A* virus. This virus-specific network also consists of a large component and a large number of small components, all of small average path length, although the number of small components is smaller and the average path length is larger than in the whole virus-host protein–protein interaction networks.

**Table 4:** Structure of the *Influenza A* virus-host protein–protein interaction networks.

**Network**	**Nodes**	**Edges**	**Components**			**Average path length**
			**Number**	**Size**	**Count**	
VirusMentha	563	1,325	5	2	4	1.562567
IntAct	1,737	4,141	9	2	5	1.479075
				4	1	
				6	1	
				1,714	1	
VirHostNet	2,620	7,921	2	118	1	2.753600
HPIDB	3,230	10,920	7	2	2	1.719102
				4	1	
				6	1	
				118	1	
				3,095	1	
Viruses.STRING	4,183	6,831	1	4,183	1	3.161478
PHISTO	2,943	10,416	5	2	2	1.735121
				2,931	1	
HVIDB	3,215	11,408	6	2	1	1.782689
				4	1	
				11	1	
				3,192	1	

### 2.5. Overlap of the Datasets

Most of the databases contain interactions derived from literature curation and from the other databases and thus, their overlap in terms of common proteins and interactions could be expected to be large. However, the overlap of each pair of datasets is rather small, especially with Viruses.STRING: only 35 of the 534 interactions in EBI-GOA-nonIntAct, 235 of the 5,157 interactions in BioGRID, 4,424 of the 10,625 interactions in VirusMentha, 3,801 of the 22,677 interactions in IntAct, 79 of the 28,132 interactions in VirHostNet, 306 of the 33,752 interactions in HPIDB, 4,669 of the 39,010 interactions in PHISTO, and 4,665 of the 40,132 interactions in HVIDB are also in the Viruses.STRING dataset.

The overlap among each three or more generic datasets is even smaller. For example, while 8,505 of the 43,944 interactions in VirusMentha, IntAct, and HPIDB are shared by the three datasets, only 3,617 of the 281,942 interactions in VirusMentha, IntAct, HPIDB, and Viruses.STRING are shared by the four datasets, only 1,180 of the 285,650 interactions in VirusMentha, IntAct, VirHostNet, HPIDB, and Viruses.STRING are shared by the five datasets, and only 38 of the 289,406 interactions in BioGRID, VirusMentha, IntAct, VirHostNet, HPIDB, and Viruses.STRING are shared by the six datasets. Further, none of the 289,753 interactions in EBI-GOA-nonIntAct, BioGRID, VirusMentha, IntAct, VirHostNet, HPIDB, and Viruses.STRING are shared by the seven generic datasets.

**Figure 3:**
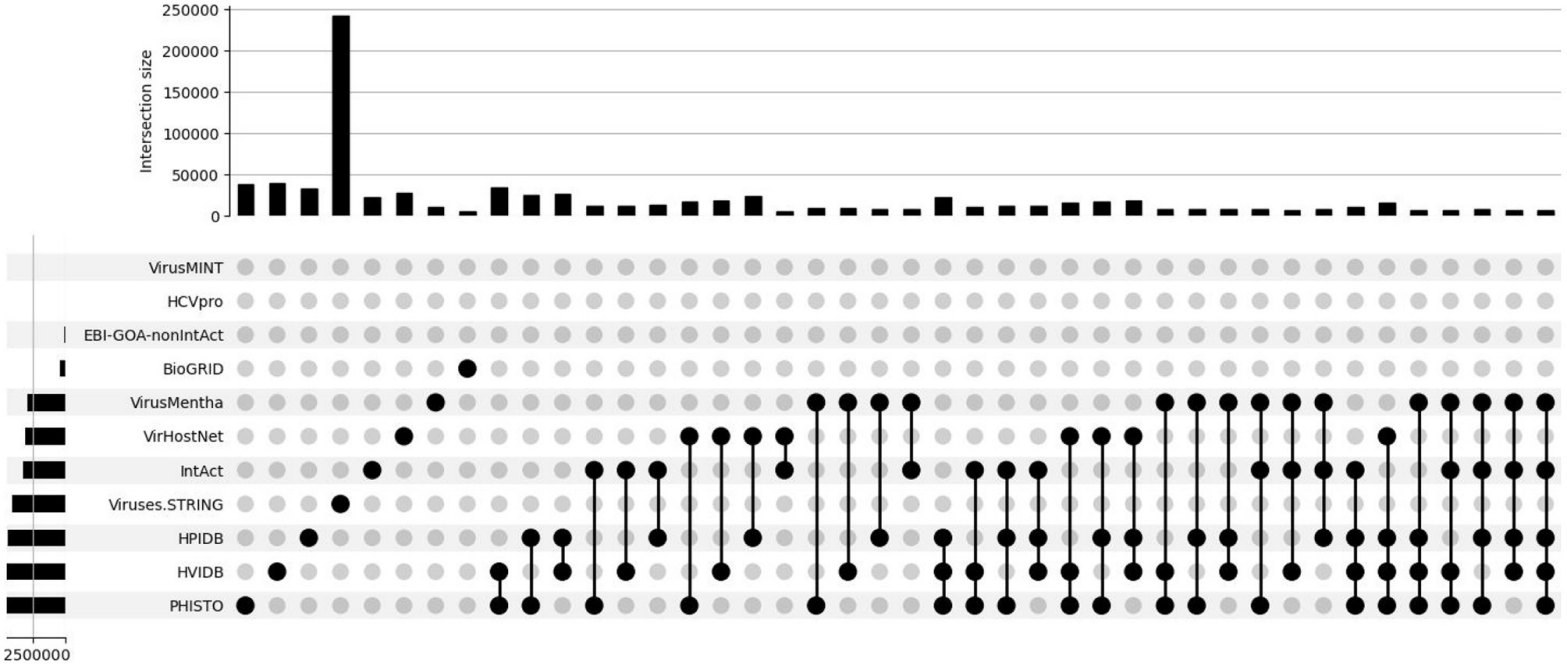
Overlap of the virus-host protein–protein interaction databases.

**Figure 4:**
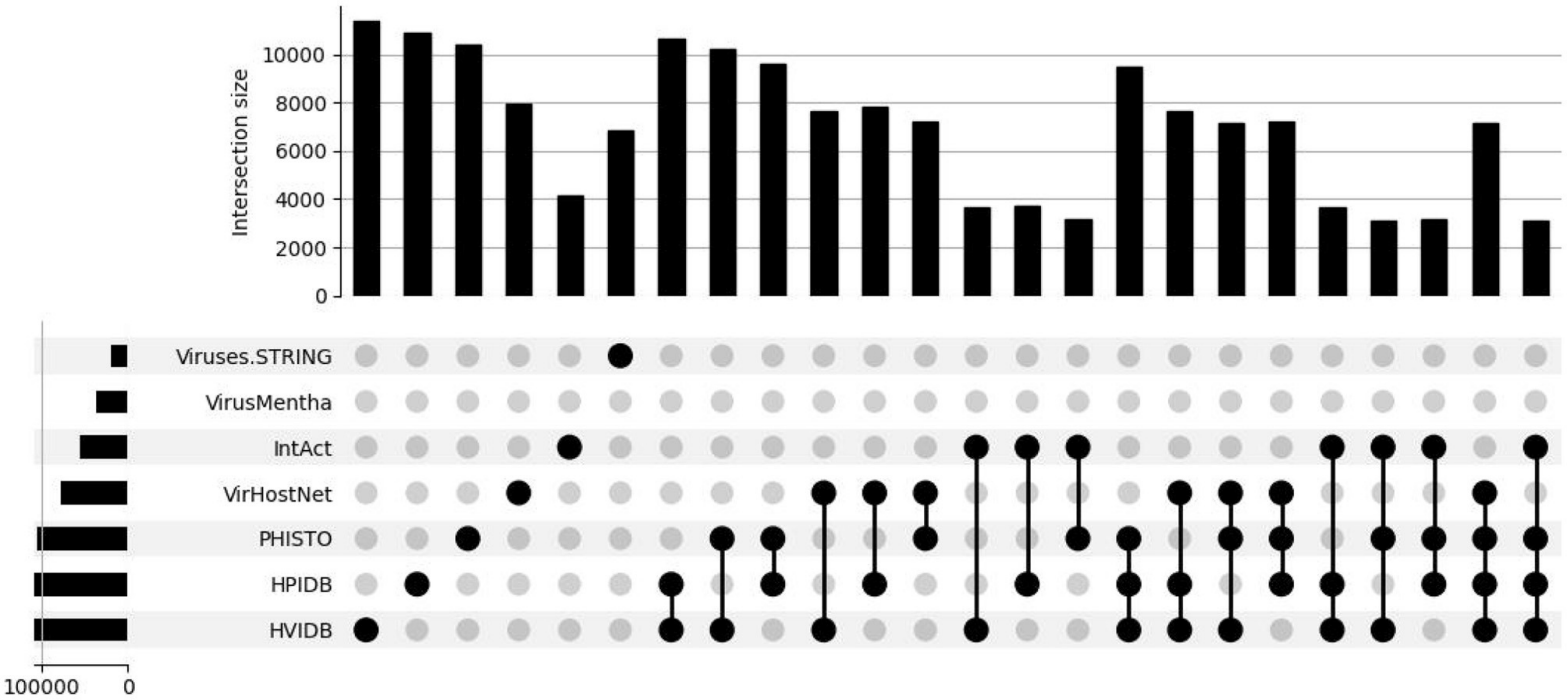
Overlap of the virus-host protein–protein interaction databases for the *Influenza A* virus.

This is all summarized in the set intersection diagram shown in Figure 3, which were obtained using a Python implementation of the UpSet tool (Lex et al., 2014). The overlap across the datasets is also small in the virus-host protein–protein interaction networks for the *Influenza A* virus, as shown in the set intersection diagram in Figure 4.

**Figure 5:**
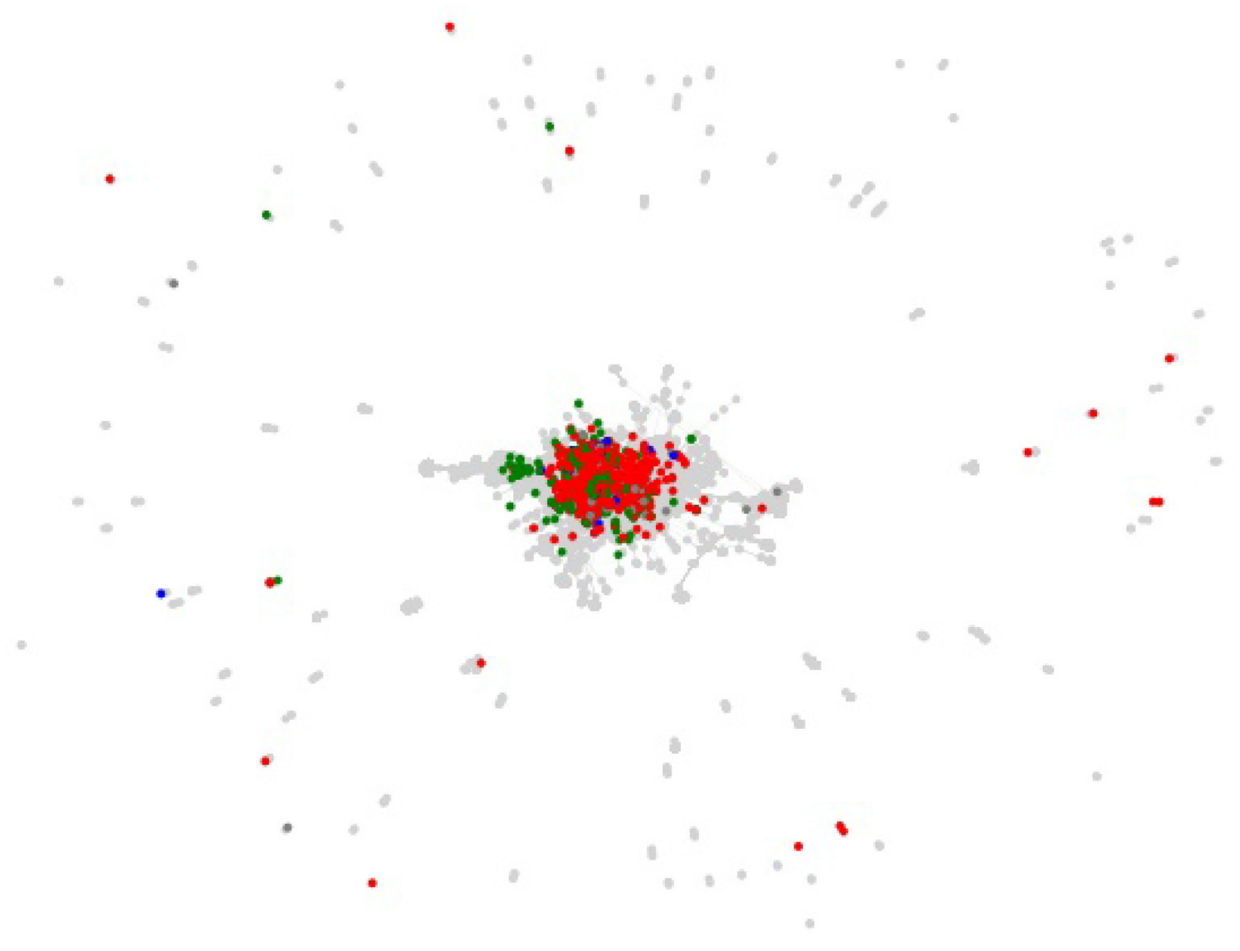
Structure of the virus-host protein–protein interaction network for the Viruses.STRING dataset and overlapping proteins and interactions in the EBI-GOA-nonIntAct, BioGRID, VirusMentha, IntAct, VirHostNet, HPIDB, HCVpro, VirusMINT, PHISTO, and HVIDB datasets (gray). Proteins overlapping only with IntAct are shown in red, proteins overlapping only with VirHostNet in green, and proteins overlapping only with BioGRID in blue.

**Figure 6:**
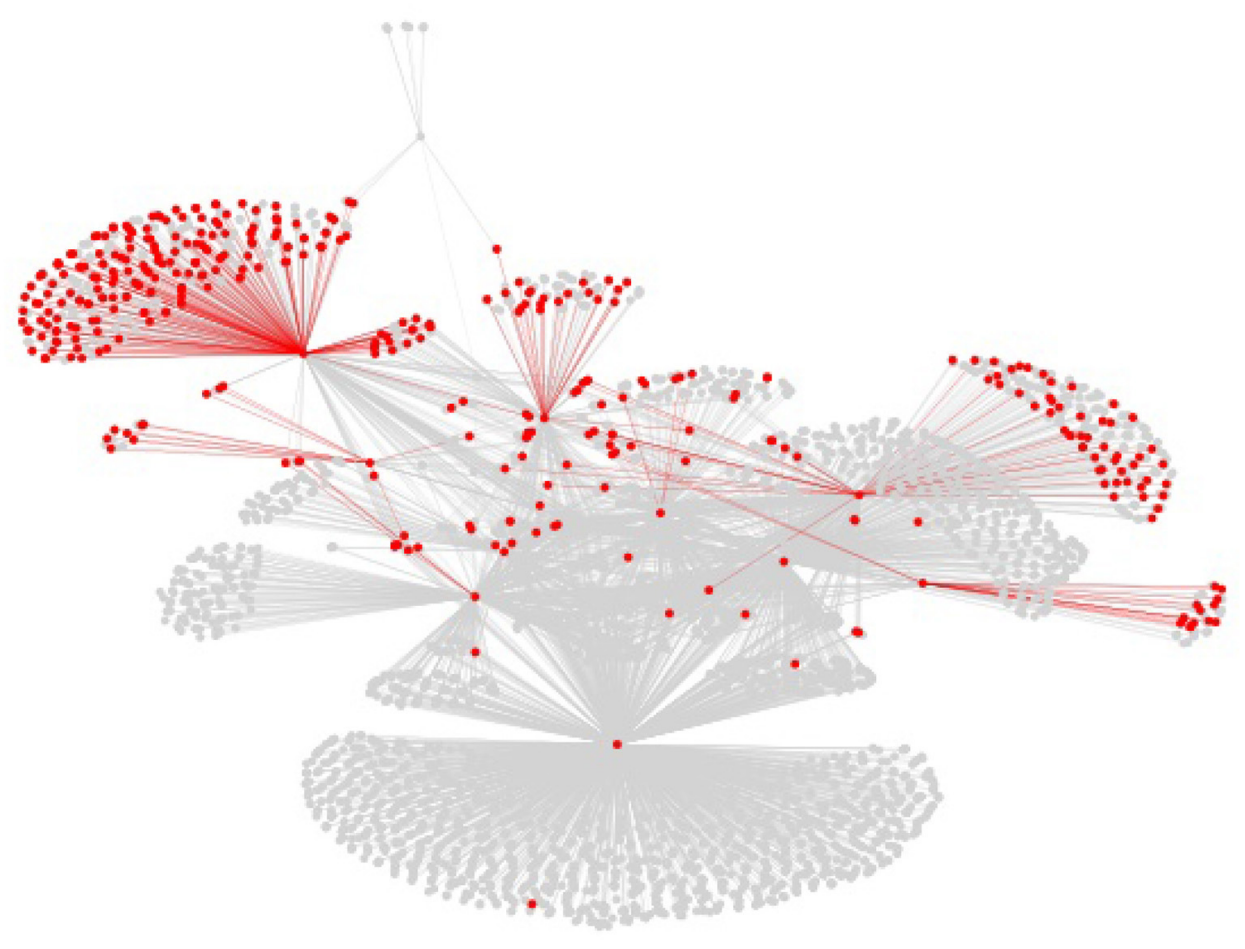
Structure of the virus-host protein–protein interaction network for the *Influenza A* virus in the Viruses.STRING dataset (gray) and overlapping proteins and interactions in the VirusMentha dataset (red).

The centrality of proteins and interactions in the virus-host protein–protein interaction networks can also be studied by means of topological measures, in order to establish whether the networks overlap on central or on peripheral proteins and interactions. For example, the centrality of a virus-host protein–protein interaction can be measured by means of the betweenness centrality of the corresponding edge in the virus-host protein–protein interaction network, which is the sum of the fraction of all-pairs shortest paths in the network that contain the edge (Brandes, 2008). However, visual inspection of the virus-host protein–protein interaction networks, as shown in Figure 5 for the Viruses.STRING dataset along with all the other datasets, suffice to determine that they overlap on peripheral, as opposed to central, interactions. The overlap on peripheral proteins and interactions is even more clear in the virus-host protein–protein interaction networks for the *Influenza A* virus in the Viruses.STRING and VirusMentha datasets, shown in Figure 6.

## 3. Discussion

Central to the comparative review of the available virus-host protein–protein interaction database resources is the mapping of the virus and host protein identifiers used in each of the databases to unique proteins identifiers. The reader may be familiar with the good old six-symbol unique identifiers found in the UniProtKB-AC database (The UniProt Consortium, 2017). There are about 30 million 6-symbol and about 200 million 8-symbol identifiers stored therein now, what comes as a surprise since unique identifiers made up of six letters and digits would suffice to store over two billion proteins. Nevertheless, the comparative analysis of virus-host protein–protein interaction databases requires mapping proteins to unique protein identifiers such as those in UniProtKB-AC.

While some of the databases include such a mapping, it is in general neither complete nor up-to-date. The mapping problem is not trivial, as the virus and host protein identifiers used in the databases do not always map to unique proteins identifiers. Moreover, some of the databases even include proteins annotated to multiple organisms, such as HVIDB, which has 552 unique proteins in 10,689 interactions annotated to multiple organisms, often along the same lineage. Thus, the identifier mapping problem can only be partially solved, and about 25% of the proteins in the generic, virus-specific, and host-specific databases had to be discarded because they could not be mapped to unique UniProtKB-AC identifiers.

Overall, the generic, virus-specific, and host-specific databases have very good search and visualization facilities. However, when it comes to downloading protein–protein interaction data for further use, most of the databases have their own protein identifiers and include only partial, if any, unique mappings to UniProtKB-AC. Indeed, once the protein identifiers in the various databases have been mapped to UniProtKB-AC identifiers, the resulting datasets have a rather small overlap. For example, while 14.27% of the interactions in BioGRID, 31.84% of the interactions in EBI-GOA-nonIntAct, 61.90% of the interactions in IntAct, 84.60% of the interactions in VirHostNet, and 84.71% of the interactions in VirusMentha are also found in HPIDB, only 4.55% of the interactions in BioGRID, 5.30% of the interactions in VirHostNet, 6.55% of the interactions in EBI-GOA-nonIntAct, 12.41% of the interactions in HPIDB, 16.76% of the interactions in IntAct, and 41.64% of the interactions in VirusMentha are also found in Viruses.STRING.

Further, the structural analysis of the virus-host protein–protein interaction networks showed that the databases overlap mostly on peripheral interactions, and the central interactions in the networks are not shared among the databases. This comes as a surprise, because essential proteins are known to have higher centrality in a protein–protein interaction network than the network average (Jeong et al., 2001; Raman et al., 2014) and thus, central proteins and interactions are more widely studied and more likely to be reflected in virus-host protein–protein interaction databases than peripheral proteins and interactions. The structural analysis of the virus-host protein–protein interaction network for the *Influenza A* virus, on the other hand, showed that it has a smaller number of small components and a larger average path length than the other virus-host protein–protein interaction networks, which can be explained by *Influenza A* being a widely studied virus, with a larger fraction of the virus-host protein–protein interactions reflected in the databases.

## Data Availability Statement

The datasets generated for this study (virus-host protein-protein interactions) are available in the Supplementary Material.

## Author Contributions

The author confirms being the sole contributor of this work and has approved it for publication.

## Funding

This research was partially supported by the Spanish Ministry of Science and Innovation, and the European Regional Development Fund, through project PID2021-126114NB-C44 (FEDER/MICINN/AEI).

## Conflict of Interest

The author declares that the research was conducted in the absence of any commercial or financial relationships that could be construed as a potential conflict of interest.

## Publisher's Note

All claims expressed in this article are solely those of the authors and do not necessarily represent those of their affiliated organizations, or those of the publisher, the editors and the reviewers. Any product that may be evaluated in this article, or claim that may be made by its manufacturer, is not guaranteed or endorsed by the publisher.
